# Supplementary material for: Bromodomain and Extraterminal Protein Inhibitor, Apabetalone (RVX-208), Reduces ACE2 Expression and Attenuates SARS-Cov-2 Infection In Vitro
Source: Biomedicines. 2021 Apr 18;9(4):437. doi: 10.3390/biomedicines9040437 (PMC8072876; doi:10.3390/biomedicines9040437)
Supplement: Supplementary file 1 [file biomedicines-09-00437-s001.zip › biomedicines-1173899-supplementary.pdf]

Table S1

Table S1: Characteristics of Primary Human Hepatocyte Donors

Donor #	Sex	Race	Smoking	Cause of Death	Age	BMI	Medications
**Donor 1**	M	Caucasian	no	MVA	3	15	None reported
**Donor 2**	F	Caucasian	yes	stroke	47	22	None reported
**Donor 3**	F	Caucasian	yes	n/a	70	21	None reported

n/a = not available. MVA = motor vehicle accident. BMI = body mass index (kg/m^2)

Fig. S1

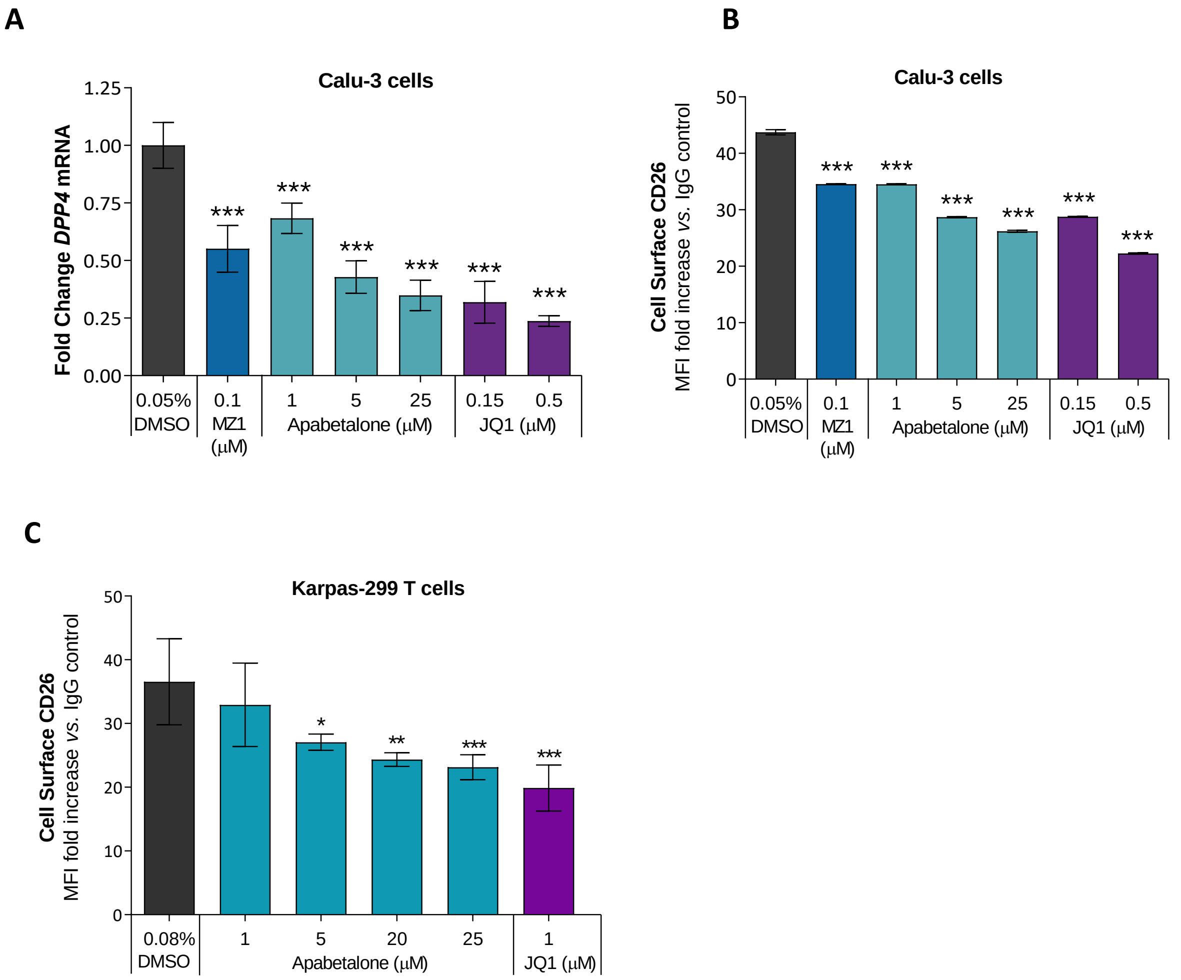

Fig. S1: Evaluation of *DPP4* gene expression and CD26 protein levels in Calu-3 lung epithelial cells (A-B) or Karpas-299 T cells (C) following 48 h treatment with the indicated BETi compounds or vehicle alone (DMSO). *DPP4* mRNA levels were quantified by real-time PCR using cyclophilin as a reference gene. Transcript levels of *DPP4* are presented as fold change relative to DMSO treated cells (A). Flow cytometric analysis of cell surface expression of DPP4 (CD26) in Calu-3 cells (B) or Karpas-299 cells (C) was estimated as MFI fold increase *vs.* IgG control. Experiments were performed at least 3 independent times. *p<0.05, **p<0.01, ***p<0.001, one-way ANOVA followed by Dunnett's multiple comparison test

Fig. S2

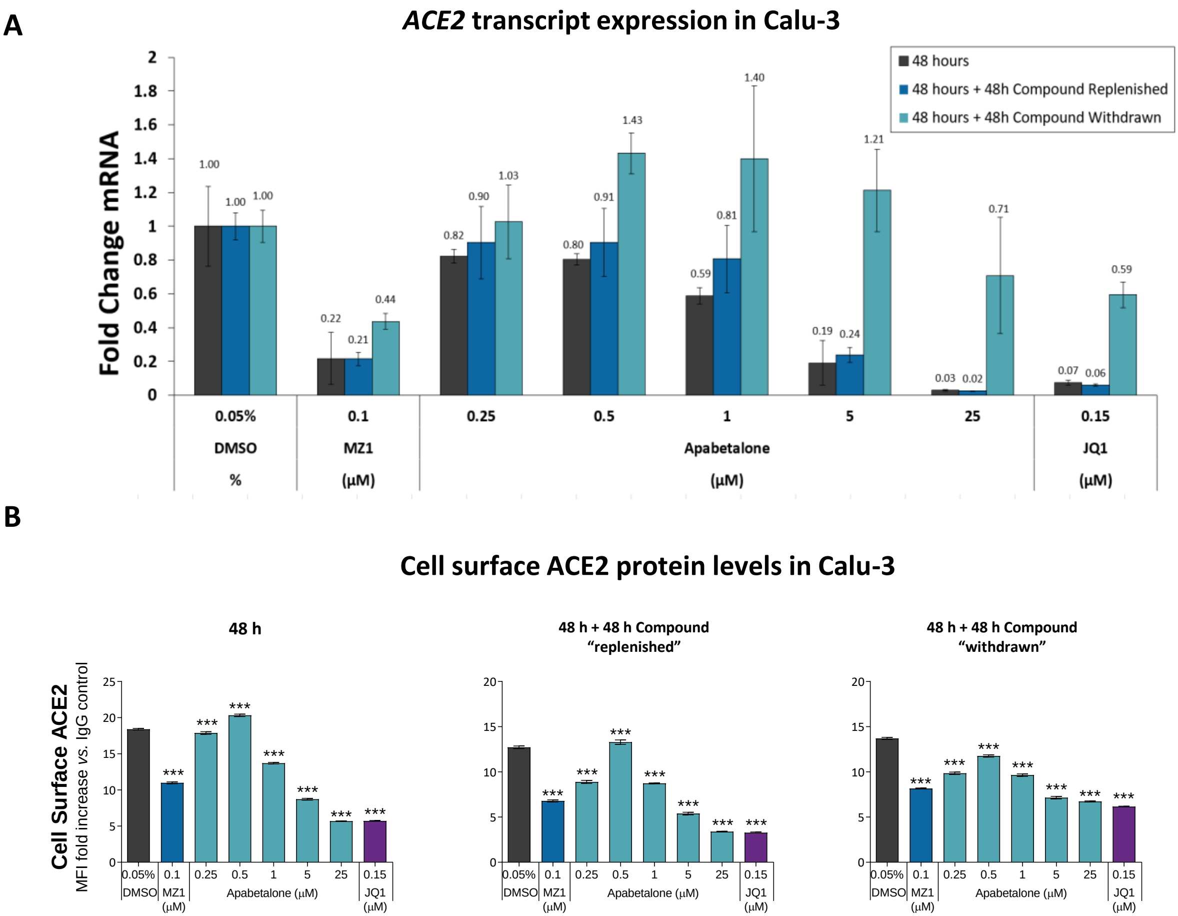

Fig. S2: Comparison of *ACE2* gene expression and cell surface protein levels in BETi treated Calu-3 cells. Calu-3s were pre-treated with the indicated BETi for 48 h, washed and then replenished with complete medium +/- BETi for another 48 h. Cells were harvested at 48 h, or 96 h (BETi replenished or withdrawn) and assessed for *ACE2* mRNA levels by real-time PCR (A) or cell surface ACE2 protein levels by flow cytometry (B). Experiments were performed at least 3 independent times. *p<0.05, **p<0.01, ***p<0.001, one-way ANOVA followed by Dunnett's multiple comparison test.

Fig. S3

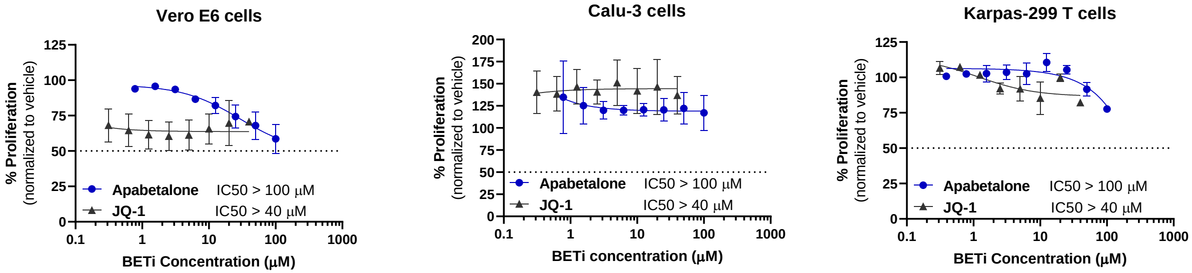

Apabetalone dose range: 0.781 - 100 μM
JQ1 dose range: 0.3125 - 40 μM

Apabetalone dose range: 0.78125 - 100 μM
JQ1 dose range: 0.3125 - 40 μM

Apabetalone dose range: 0.3906 - 100 μM
JQ1 dose range: 0.3125 - 40 μM

Fig. S3: Effect of BETi on cell viability in the absence of SARS-CoV2 infection. Effects of BETi following 48 h treatment on proliferation of un-infected Vero E6, Calu-3 and Karpas-299 T cells was evaluated by MTS assay (n=3/BETi concentration tested). IC50 concentrations were not reached at the tested concentrations as indicated by the dotted line.
